# Detection of *Human parvovirus B19* (HPVB19) in serum samples from fever-rash ill individuals during the rubella outbreak (2005) in Bulgaria

**DOI:** 10.1080/13102818.2014.967746

**Published:** 2014-10-29

**Authors:** Andon Toshev, Stefka Ivanova, Valentina Kovaleva, Lyubena Andonova, Zafira Mihneva

**Affiliations:** ^a^ Department of Biology, Medical University of Sofia, Sofia, Bulgaria; ^b^ NCIPD, National Reference Laboratory of ‘Measles, Mumps, Rubella’, Sofia, Bulgaria; ^c^ Virology Unit, Research Center of Military Epidemiology and Hygiene, Military Medical Academy, Sofia, Bulgaria; ^d^ Specialized Hospital for Infectious and Parasitic Diseases ‘Prof. Ivan Kirov’, Sofia, Bulgaria

**Keywords:** *parvovirus B19*, fever-rash infection, HPVB19-IgM antibodies, ELISA, HPVB19-DNA, PCR

## Abstract

The present study aimed to determine the involvement of the *parvovirus B19* (HPVB19) as an etiological agent in individuals with fever-rash infections but not infected with rubella during the rubella outbreak (2005) in Bulgaria. A total of 194 serum samples with negative results for measles and rubella-specific IgM antibodies were tested in the National Reference Laboratory. The individuals aged 5–52 years (mean age 17.2 ± 10.15) were divided into four age groups: 5–14; 15–24; 25–34; and 35+ years old. Serological (indirect enzyme immunoassay – EIA) and molecular (extraction and detection of HPVB19-DNA) methods were used. A genotyping assay of the NS1-PCR product was proceeded with the MfeI restriction enzyme. Out of the total number of samples, 95 samples (48.97%) tested positive for HPVB19-IgM and 109 (56.18%) for HPVB19-DNA. The results from the genotyping assay revealed that genotype 1 (prototype B19) was dominant in 106 from 109 samples (97.25%), while genotype 3 (prototype V9) was detected in only 3 (2.75%). Subjects whose sera tested positive for IgM and had a positive PCR result formed a group that was most frequently linked (in 40% of cases) to acute infection. The highest prevalence was established in the group of the school-age children (5–14 years). The combined application of serological and molecular methods confirms the etiological role of HPVB19, and including virus genotyping, confirms the involvement of HPVB19 in the etiological palette of febrile rash diseases and provides a correct differential diagnostic approach.

## Abbreviations

*HPVB19:* *Human parvovirus B19*
*ELISA:* enzyme-linked immunosorbent assay
*PCR:* polymerase chain reaction
*DNA:* deoxyribonucleic acid

## Introduction

Following the use of mass prophylaxis of vaccine-preventable infections a large percentage of rash-fever illnesses occur in a clinically atypical way. This requires an enhanced epidemiological surveillance and extending the differential spectrum of the diagnostic testing.

*Parvovirus B19* (HPVB19) is the etiological agent of *Erythema infectiosum*, the so-called fifth disease. The present study is retrospective and intended as a pilot research for the country. Its results support the inclusion of the HPVB19 testing of serum samples in the routine laboratory practice and the differential diagnostic algorithm in fever-rash individuals.

HPVB19 is a human pathogen classified in the *Parvoviridae* family (genus *Parvovirus*), and is isolated in 1975 by the team of the Australian virologist Cossart during the screening of blood donor samples for hepatitis B.[1] In 1995, due to the accumulated genetic and functional differences between HPVB19 and the other members of the *Parvovirus* genus, the virus was classified into the *Erythrovirus* genus [2,3] with a main feature – the ability to replicate in human bone marrow cells (erythroid progenitor cells) and to infect a wide range of cells that have receptor blood group P antigen on their surface. Based on the phylogenetic analysis (2010) of the DNA sequences of NS1-VP1unique junction region, *Human erythrovirus B19* was subdivided into three genotypes: B19V-related viruses corresponding to genotype 1 (prototype strain Au), A6-related viruses corresponding to genotype 2 (prototype strains Lali and A6) and V9-related viruses corresponding to genotype 3 (prototype strains V9 and D 91.1), with no impact on the clinical course of the HPVB19.[4]

The virus is widely spread with a winter–spring seasonality and affects groups of children as small epidemics and in about 20% of the infected runs as an asymptomatic infection.

The registered seroprevalence in children under 5 years is 2%–10%, 15%–40% in young adolescents (10–19 years old), 45%–60% of adults over 20 years, and more than 75% of adults over 60.[5]

The clinical aspect of the viral infection is represented by a very diverse pathology resulting from the tropism and route of transmission (airborne, parenteral and transplacental) of HPVB19.[6] The infection occurs as a transient self-limiting disease but it can also present as severely as a fulminant form depending on the state of the immune system of the infected individual. Besides being an etiological agent of a rash illness, HPVB19 also causes aplastic anaemia and may be an important pathogen in individuals with hematologic disorders, for seronegative pregnant women, immunocompromised patients, etc.

Modern diagnostics of HPVB19 infection usually includes detection of HPVB19-IgM and HPVB19-IgG antibodies in the blood by an ELISA test and amplification of HPVB19-DNA (NS1 conservative region) from blood or tissue samples (bone marrow aspirate, amniotic and synovial fluids, biopsy materials, etc.) by hybridization methods and PCR techniques.[7]

The objective of this study was to determine the prevalence of HPVВ19 as an etiological agent in individuals with fever-rash infections that were not infected with measles and rubella during the rubella outbreak in 2005.

## Materials and methods

### Samples and study group

Our retrospective study included 194 serum samples from all over the country, received at the National Reference Laboratory (NRL) during the rubella outbreak in 2005. All samples tested negative for measles and rubella-specific IgM antibodies. The individuals, aged 5–52 years (mean age 17.2 ± 10.15) were divided into four age groups: 5–14; 15–24; 25–34; and over 35 years old.

### Enzyme immunoassay

Indirect ELISA tests for the detection of specific measles, rubella and *parvovirus B19* IgM antibodies (Enzygnost Anti-Measles Virus/IgM, SIEMENS; Enzygnost Anti-Rubella Virus/IgM, SIEMENS; *parvovirus B19* IgM, *recom*Well) were used according to the manufacturers’ instructions.

### Molecular methods

Extraction of viral HPVB19-DNA was done with the commercially available kit NucleoSpin® Blood according to the instructions provided by the manufacturer.

The PCR test was performed in a total volume of 25 μl using the following chemical composition: AmpliTaq Gold PCR Master Mix diluted to a working concentration with nuclease-free water (18 μl); 1 μl from each of the primers e1905f and e1987r; and 5 μl of DNA template (Table 1).

**Table 1.** HPVB19-PCR – parameters (according to Servant et al. [4]).

Gene conservative	Primer ^a^		Amplification profile		
region	name	Nucleotide sequence (5′– 3′)	Cycle	Temperature (°C)	Time
NS1 NS1	e1905f e1987r	TGCAGATGCCCTCCACCCA GCTGCTTTCACTGAGTTCTTC	1 cycle	94 °C	6 min
				94 °C	30 sec
			5 cycles	55 °C	1 min
				72 °C	1 min
				94 °C	30 sec
			45 cycles	60 °C	30 sec
				72 °C	30 sec
			Final elongation	72 °C	7 min
			Hold	4 °C	

Note: ^a^f: foreword; r: reverse primer.

Discrimination between the HPVB19 viral genotypes (B19 and V9) was done by MfeI (MunI) restriction enzyme of the NS1-PCR product according to the manufacturer's digestion protocol. Briefly, following PCR amplification the resulting product was directly digested by mixing 10 μl from the PCR reaction composition with 18 μl of nuclease free water, 2 μl of 10X Buffer G and 2 μl from the restriction enzyme MfeI. After incubation at 37 °C for 2 hours and thermal inactivation at 65 °C for 20 min, an electrophoresis in 2% and 3% agarose gels was performed to visualize the NS1-PCR products. DNA ladders with a molecular marker size of 25 bp (25–700 bp) and 50 bp (50–1000 bp) were included to verify the size of the amplicons (PCR product with a size of 103 bp and restriction fragments of 36 and 67 bp).

## Results and discussion

The present study included individuals from all over the country with serum samples taken to NRL during 2005 (the year with increased incidence of rubella in Bulgaria), clinically diagnosed as ‘rubella’ and/or as ‘measles’ and showing negative IgM serologic results for both infections. These samples were then tested for HPVB19.

The clinical manifestations of rubella and parvovirus infection are quite similar (prodromal period, confluent erythema rash, joint pain, some post-infectious complications, etc.). In this regard, parvovirus infections are more frequently confirmed during rubella outbreaks in comparison to other diseases showing similar types of rash. These findings are also confirmed by other researchers.[8,9]

Patients in the study were distributed by age into four groups, according to the known circulation data for the two viruses (*parvovirus B19* and rubella) among the population: school-age children, adolescent, young adults and mean-age individuals.

**Figure 1.**
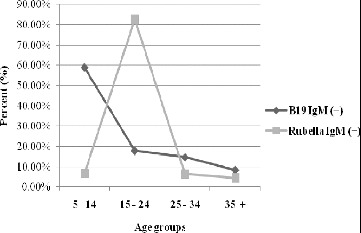
Distribution of the positive results for HPVB19 and rubella IgM antibodies according to the defined age groups (%).

Due to the implementation of specific rubella prophylaxis in the country (as a monovalent vaccine and as part of the MMR vaccine since 1988 and 2001, respectively), the rubella infection is mostly observed in senior age groups. The obtained results for 2005 in the groups defined by age were compared in terms of the serological markers (positive specific IgM antibodies) for *Erythema infectiosum* and rubella as shown in Figure 1.

**Figure 2.**
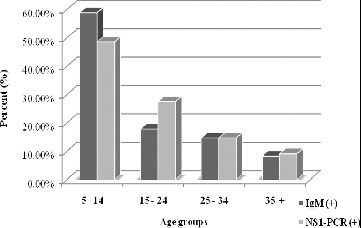
HPVB19-IgM and HPVB19-DNA positive results among the tested age groups (%).

All 194 samples negative for specific measles and rubella IgM antibodies were tested for specific HPVB19-IgM and HPVB19-DNA, which were the most frequently used diagnostic markers. Positive results for HPVB19-IgM were found in 95 out of 194 (48.97%) and for HPVB19-DNA in 109 out of 194 (56.18%) of the tested sera. The prevalence of positive results (in percentage) in the age groups is presented in Figure 2.

The group of the school-age children (5–14 years) was found to be the most affected one as measured by the diagnostic marker HPVB19-IgM (56 out of 95, 58.9%) and by HPVB19-DNA (53 out of 109, 48.6%), followed by the group of adolescents (15–24 years), wherein an etiologically demonstrated infection was primarily revealed by a higher detection of viral DNA than of the specific IgM antibodies (27.50% and 17.90%, respectively). This could be explained by a visit to healthcare specialists later in the course of the disease; older age; an atypical onset of infection (flu-like illness, arthropathy, anaemic syndrome, etc.); viral reinfection when specific IgM antibodies rapidly disappear or are undetectable by most tests.

The least-affected group was that of patients over 35 years of age which indicates the increased seroprevalence of protective specific HPVB19-IgG antibodies among such individuals.

**Figure 3.**
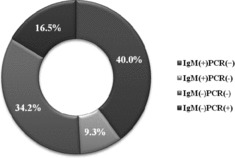
Combined results from the two diagnostic methods for the detection of HPVB19 infection (%).

The combined application of serological and molecular methods confirmed the etiological participation of HPVB19 and is demonstrated in Figure 3. The group of patients with both IgM and PCR positive results typically reveals the picture of an acute HPVB19 infection (40%). Healthy individuals after a recent HPVB19 infection show viral blood titres as high as 10^12^ gEq/ml which may remain at levels of about 10^3^–10^5^ for months and even few years following the initial infection.[10]

Patients in the group with negative IgM and positive PCR results (16.5%) reveal the convalescent period, when HPVB19-IgM antibodies cannot be detected, but HPVB19-DNA is still present in the serum samples. For example, low levels of HPVB19-DNA or DNA fragments may persist in sera for many months following a recent infection and for years in other tissues.

This study documented that the combination of positive HPVB19-IgM and negative HPVB19-PCR results (9.3%) was rarest. It may be related to specific mutations in the conservative NS1 region. Such mutations could be suspected in the primer hybridization regions which does not allow for the proper use of the primer pairs.

The use of other serological markers for laboratory diagnosis such as HPVB19-IgG antibodies, the IgG avidity test as well as HPVB19-DNA detection is a useful approach for the testing of immunodeficient persons, who might not be able to produce specific IgM antibodies or when the virus is only present in different body depots.[11]

**Figure 4.**
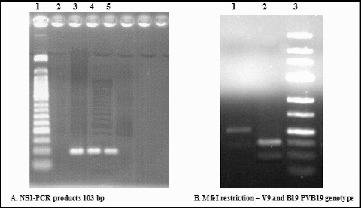
Electrophoresis in 2% and 3% agarose gels. (A) Lane 1: 50 bp molecular marker; Lane 2: negative control; Lanes 3, 4 and 5: samples with a positive result. (B) Lane 1: V9 DNA (no cleavage); Lane 2: HPVB19-DNA (two fragments with a size of 36 and 67 bp); Lane 3: 25 bp molecular marker.

The NS1-PCR assay was subsequently used to screen clinical samples for the presence and typing of *parvovirus B19*/*erythrovirus B19* DNA. MfeI restriction technique of the 103 bp NS1-PCR product allowed a clear distinction between the B19 virus DNA (resulting in two fragments of 36 and 67 bp) and the V9 DNA (no cleavage), as it is also shown in Figure 4.

More data concerning the circulation of HPVB19 genotypes in the country are forthcoming and will be available following a future sequence analysis of the NS1-VP1 unique region which is considered suitable for phylogenetic studies and also includes information on the three HPVB19 viral proteins.

The prevalence of B19- and V9-type viruses was unequally distributed between the different patients. The retrospective study showed a wider distribution and dominance of genotype 1 (prototype: *parvovirus B19*) in 106 out of 109 (97.25%) samples, while genotype 3 (prototype: isolate V9) was detected in as little as 3 out of 109 (2.75%) patient's sera. Other authors [12] report similar findings in a survey of HPVB19 viral spread in Europe and suggested that genotype 1 replaced genotype 2 which was circulating in the second half of the last century. Genotype 3 has recently been detected in France,[4] UK [13] and Germany.

## Conclusions

The laboratory diagnosis of НРVВ19 combining serological (HPVB19-IgM ELISA) and molecular (HPVB19-DNA PCR) methods together with virus genotyping for the confirmation of НРVВ19 involvement as an aetiological agent of febrile rash diseases was introduced for the first time in Bulgaria.

Among the countries which have entered in the elimination phase for vaccine-preventable infections such as measles and rubella, the better knowledge on the epidemiology of *parvovirus B19* may help clinicians with the differential diagnosis of HPVB19 clinical manifestations. This conclusion is particularly adequate during inter-epidemic periods when the majority of reported cases need to follow a correct diagnostic approach and surveillance protocols.

The detection of НРVВ19 is of particular importance for the monitoring of individuals in risk groups like women in childbearing age, medical staff, individuals with haematological disorders, and those with immunodeficiency.
